# Predicting mortality and hospitalization of older adults by the multimorbidity frailty index

**DOI:** 10.1371/journal.pone.0187825

**Published:** 2017-11-16

**Authors:** Yao-Chun Wen, Liang-Kung Chen, Fei-Yuan Hsiao

**Affiliations:** 1 Graduate Institute of Clinical Pharmacy, College of Medicine, National Taiwan University, Taipei, Taiwan; 2 Health Data Research Center, National Taiwan University, Taipei, Taiwan; 3 Institute of Public Health, School of Medicine, National Yang-Ming University, Taipei, Taiwan; 4 Aging and Health Research Center, National Yang-Ming University, Taipei, Taiwan; 5 Center for Geriatrics and Gerontology, Taipei Veterans General Hospital, Taipei, Taiwan; 6 School of Pharmacy, College of Medicine, National Taiwan University, Taipei, Taiwan; 7 Department of Pharmacy, National Taiwan University Hospital, Taipei, Taiwan; Cardiff University, UNITED KINGDOM

## Abstract

**Background:**

Existing operational definitions of frailty are personnel-costly and time-consuming, resulting in estimates with a small sample size that cannot be generalized to the population level. The objectives were to develop a multimorbidity frailty index using Taiwan’s claim database, and to understand its ability to predict adverse event.

**Methods:**

This is a retrospective cohort study. Subjects aged 65 to 100 years who have full National Health Insurance coverage in 2005 were included. We constructed the multimorbidity frailty index using cumulative deficit approach and categorized study population according to the multimorbidity frailty index quartiles: fit, mild frailty, moderate frailty and severe frailty. The multimorbidity frailty index included deficits from outpatient and inpatient diagnosis. Associations with all-cause mortality, unplanned hospitalization and intensive care unit admission were assessed using Kaplan-Meier curves and Cox regression analyses.

**Results:**

The multimorbidity frailty index incorporated 32 deficits, with mean multimorbidity frailty index score of 0.052 (standard deviation = 0.060) among 86,133 subjects included. Compared to subjects in fit category, subjects with severe frailty were associated with a 5.0-fold (adjusted hazard ratio, aHR 4.97; 95% confidence interval, 95% CI 4.49–5.50) increased risk of death at 1 year after adjusting for age and gender. Subjects with moderate frailty or mild frailty was associated with 3.1- (adjusted HR 3.08; 95% CI 2.80–3.39) or 1.9- (adjusted HR 1.86; 95% CI 1.71–2.01) folds increased risk, respectively.4.49–5.50). The risk trend of unplanned hospitalization and intensive care unit admission is similar among the study population. Besides, the association between the frailty categories and all three outcomes was slightly stronger among women.

**Conclusion:**

The multimorbidity frailty index was highly associated with all-cause mortality, unplanned hospitalization and ICU admission. It could serve as an efficient tool for stratifying older adults into different risk groups for planning care management programs.

## Introduction

As society ages, a core focus of healthcare providers and policy makers is to identify risk factors that increase the vulnerability of older adults to adverse clinical outcomes. A well-recognized geriatric syndrome, frailty, which is characterized by impaired homeostasis and decreased physiological reserve, has been linked to morbidity and premature mortality in older people.[1, 2] As current evidence suggests that frailty might be reversible with certain interventions[3, 4], the identification of frailty is thus crucial to prevent further decline in health outcomes among older people and to help highlight areas into which clinicians and policy-makers can place efforts.

A number of operational definitions of frailty have been developed to fulfill such needs[5, 6]. However, these operational definitions vary and can include different aspects of health-related measurements such as physical state, cognition, and social relations and support. Questions thus remain on how effective the definitions are at estimating the prevalence of frailty and its association with adverse clinical outcomes. In addition, some of these operational definitions are personnel-costly and time-consuming, resulting in estimates with a small sample size that cannot be generalized to the population level.[6, 7]

A large claims database with abundant healthcare information, particularly a nationwide claims database, thus could be a good option available for estimating the real-world prevalence of frailty and its association with adverse outcomes. However, such an approach has never been implemented. We aimed to develop a multimorbidity frailty index (mFI) using Taiwan’s National Health Insurance Research Database (NHIRD).[8] We further examined the ability of the developed mFI to predicting 1-, 5-, and 8-year hospitalizations and mortality.

## Methods

### Study design and study cohort

This is a cohort study using data from Taiwan’s National Health Insurance Research Database (NHIRD), a nationwide database composed of outpatient and inpatient claims for 99% of Taiwan’s population. The NHIRD has been widely used for many population-level studies, including a number of studies in geriatrics and gerontology.[9, 10] We conducted this retrospective cohort study using one subset of NHIRD, the Longitudinal Health Insurance Database (LHID), which contains claims data of one-million randomly selected beneficiaries from the Registry of Beneficiaries of the NHIRD in 2005.[11] Claims data from 2005 to 2013 for the one million beneficiaries was extracted to compose a 9-year (2005–2013) panel of claims for analysis.

The study cohort consisted of all subjects aged 65 to 100 years who had full National Health Insurance (NHI) coverage from January 1, 2005 to December 31, 2005.

### Ethical statement

The study protocol was approved by the Research Ethics Committee of the National Taiwan University Hospital (NTUH-REC-201403069W).

### Construction of the multimorbidity frailty index

We adopted the cumulative deficit approach to construct the multimorbidity frailty index (mFI). The cumulative deficit approach is one of the most commonly used models for the definition of frailty, which collectively includes variables for disease state, signs and symptoms and disability to define deficits[8]. A frailty index can be defined as a simple calculation for the presence of each deficit as a proportion of the total.[12]

For each study subject, we retrieved all diagnoses [*International Classification of Diseases*, *Ninth Revision*, *Clinical Modification* (ICD-9-CM)] recorded in the outpatient and inpatient claims of the NHIRD between January 1 and December 31, 2005. These recorded diagnoses were then used for deficit identification. Diagnoses with the same first 3 digit of code will be considered as a potential item to be included. The codes meeting the following criteria were considered the deficits included in our calculation of mFI: (i) the prevalence of diagnoses should be more than 2%; (ii) after plotting the prevalence of diagnoses among 5 age groups (65–69, 70–74, 75–79, 80–84 and ≥85 years old), the linear regression coefficients should be positive and R^2^ value should be more than 0.30; and (iii) diagnoses that reached 100% prevalence by age 65 should be excluded. These criteria were mainly based on a previous study, in which the authors developed a frailty index using medical records as the data source.[13] Additionally, to ensure the specificity of every deficit, only those who had at least 3 outpatient or 1 inpatient claims record of that specified diagnosis code were considered as having the specified deficit. For example, an older adult must have at least 3 outpatient or 1 inpatient claims record of Parkinson's disease [ICD-9-CM: 332] to be defined as having a deficit based on our definition. Noteworthy, the discrepancy between the study completed in the UK and our study is that we required the prevalence of a potential deficit to be more than 2%, while the UK study included deficits with prevalence to be more than 0.5%. The reason we set a higher cut-off is because of the concern of our high accessibility to medical care due to our National Health Insurance system.

All deficits identified were denoted in binary form, i.e., ‘1’ indicated the presence of a deficit and ‘0’ indicated the absence of a deficit. We adopted the non-weighted method[8] to develop the mFI, which was calculated as

$$Multimorbidity\;Frailty\;Index=\frac{Number\;of\;deficits\;a\;patient\;has}{Number\;of\;total\;deficit\;items}$$

The mFI was a number between zero and one, and a larger mFI represents the frailer state of the individual. Based on the deficits we identified for our subjects, we calculated their mFIs. We further categorized them into four categories according to quartiles of their mFIs: fit, mild frailty, moderate frailty and severe frailty.

### Outcomes of interest

In this study, the outcomes of interest were all-cause mortality, unplanned hospitalization and intensive care unit (ICU) admission. All-cause mortality was identified as the date of disenrollment due to death from the NHIRD.[14] Unplanned hospitalization was defined as a hospital admission after an emergency department visit.[15] ICU admission was identified as a hospital admission with use of ICU services recorded in the NHIRD. All study subjects were continuously followed from January 1, 2006 to the occurrence of each outcome or to the end of 2013, whichever came first. For the outcomes of unplanned hospitalizations and ICU admissions, subjects were censored at death if it occurred first. Pre-planned analyses were conducted to estimate how effective the developed mFI was at predicting mortality and hospitalizations at 1, 5, and 8 years after estimating the mFI.

### Statistical analysis

Numerical variables were expressed as the mean ± standard deviation (SD) and categorical variables were expressed as number and/or percentage. The Kaplan-Meier survival curve with log-rank test was used to examine the association between categories of mFI (fit, mild frailty, moderate frailty and severe frailty) and eight-year mortality and hospitalization. Bivariate and multivariate Cox proportional hazard models were used to estimate the hazard ratios (HRs) and 95% confidence intervals (95% CIs) for mortality and hospitalizations at 1, 5, and 8 years after estimating the mFI, considering mFI as the independent variable. We further included age and gender as covariates in all adjusted models. We also performed secondary analyses to see whether there were any gender differences in these associations.

All of the analyses were performed using SAS, version 9.3 (SAS Institute Inc., Cary, NC, USA). We used the ASSESS and TEST statement in PROC PHREQ to check the proportional hazards assumption and linear relationship between the log hazard and each covariate. The PROC PHREQ also provided model fit statistics and three different chi-square statistics (likelihood ratio, score and Wald tests) to address the goodness of fit issue. The statistics in our study showed that the large-sample approximations are working well and the results are trustworthy.The LOGISTIC procedure of SAS software was subsequently used to yield the area under the receiver operating characteristic (ROC) curve (*C*-statistics) and pseudo-R^2^ estimates to assess the discrimination and variability explained by the categories of mFI (fit, mild frailty, moderate frailty and severe frailty) for each outcome.

## Results

Overall, 86,133 subjects aged 65 to 100 years were included in this study. Among the study cohort, 49.82% were male, and the mean age was 73.89 years old (SD = 6.37). We identified 32 deficits that met the eligibility criteria. The prevalence of individual deficits in the study cohort and in different age groups was summarized in **S1 Table**. The mean mFI of the total population, stratified by gender and age group, is shown in **Table 1**. The distribution of mFIs was right-skewed (**S1 Fig**). The median mFI score was 0.031 (range 0.000–0.053) and the 99^th^ percentile was 0.250. We thus categorized subjects with an mFI score of 0–0.0625 as fit, 0.0625–0.125 as mild frailty, 0.125–0.1875 as moderate frailty and >0.1875 as severe frailty, respectively. The percentages for these categories were 75.06% (fit), 16.54% (mild frailty), 5.50% (moderate frailty) and 2.90% (severe frailty).

**Table 1:** Multimorbidity frailty index by gender and age group.

	Overall (n = 86,133)	Male (n = 42,914)	Female (n = 43,219)
	Mean (SD)	Mean (SD)	Mean (SD)
**65–69 (n = 28,480)**	0.037 (0.048)	0.038 (0.049)	0.037 (0.046)
**70–74 (n = 23,700)**	0.050 (0.056)	0.053 (0.060)	0.046 (0.053)
**75–79 (n = 18,765)**	0.062 (0.065)	0.067 (0.070)	0.056 (0.059)
**80–84 (n = 9,934)**	0.070 (0.071)	0.076 (0.075)	0.064 (0.065)
**≥85 (n = 5,254)**	0.070 (0.074)	0.077 (0.080)	0.064 (0.069)
**Total (n = 86,133)**	0.052 (0.060)	0.056 (0.064)	0.048 (0.056)

SD = standard deviation

**Fig 1:**
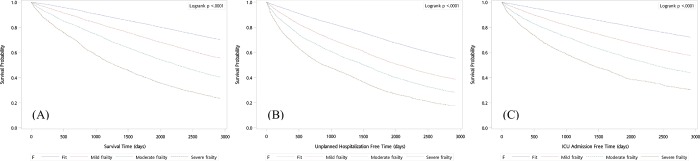
**Eight-year Kaplan-Meier survival curve for the outcome of (A) all-cause mortality, (B) unplanned hospitalization and (C) ICU admission for different frailty categories**.

With the average follow up of 6.57 (SD = 2.37) years, 30,136 deaths (34.99%) occurred among our study cohort during the study period. The results of the 8-year Kaplan-Meier survival analysis of all-cause mortality was significantly different between subjects in the four different frailty categories. Significant differences were also found for unplanned hospitalization and ICU admission (**Fig 1**). Hazard ratios of all-cause mortality, unplanned hospitalization and ICU admission at 1, 5 and 8 years increased for the mild, moderate and severe frailty categories compared with the fit category (**Table 2**). Subjects with severe frailty were associated with a 5.0-fold (adjusted HR 4.97, 95% CI 4.49–5.50) higher risk for death at 1 year after adjustment for age and gender. Subjects with moderate frailty or mild frailty were associated with a 3.1-fold (adjusted HR 3.08, 95% CI 2.80–3.39) or 1.9-fold (adjusted HR 1.86, 95% CI 1.71–2.01) higher risk for death at 1 year. The risk trend is similar with the other two outcomes although the magnitude of the risk is different. In stratification analysis, the results showed that the association between the frailty categories and all three outcomes was slightly stronger among women (**Table 3**). In ROC analysis, the mFI demonstrated moderate discrimination for the outcomes of all-cause mortality, unplanned hospitalization and ICU admission (**S2 Table**).

**Table 2:** 1-, 5- and 8-year hazard ratios for outcomes of all-cause mortality, unplanned hospitalization and ICU admission associated with different frailty categories (n = 86,133).

Outcome	Mild frailty (n = 14,244)	Moderate frailty (n = 4,741)	Severe frailty (n = 2,498)
**1-year all-cause mortality HR (95% CI)**			
Unadjusted	2.21 (2.04–2.39)	4.09 (3.72–4.50)	7.52 (6.81–8.30)
Adjusted	1.86 (1.71–2.01)	3.08 (2.80–3.39)	4.97 (4.49–5.50)
**5-year all-cause mortality HR (95% CI)**			
Unadjusted	1.76 (1.70–1.82)	2.85 (2.72–2.99)	5.00 (4.74–5.28)
Adjusted	1.46 (1.41–1.52)	2.14 (2.04–2.25)	3.28 (3.11–3.46)
**8-year all-cause mortality HR (95% CI)**			
Unadjusted	1.69 (1.64–1.74)	2.65 (2.55–2.76)	4.50 (4.29–4.71)
Adjusted	1.41 (1.37–1.45)	2.01 (1.93–2.09)	2.98 (2.84–3.12)
**1-year unplanned hospitalization HR (95% CI)**			
Unadjusted	2.08 (1.97–2.20)	3.30 (3.07–3.54)	5.29 (4.88–5.73)
Adjusted	1.91 (1.80–2.01)	2.85 (2.65–3.06)	4.28 (3.94–4.64)
**5-year unplanned hospitalization HR (95% CI)**			
Unadjusted	1.78 (1.73–1.83)	2.51 (2.40–2.62)	3.85 (3.65–4.06)
Adjusted	1.61 (1.57–1.66)	2.14 (2.05–2.24)	3.05 (2.89–3.23)
**8-year unplanned hospitalization HR (95% CI)**			
Unadjusted	1.67 (1.63–1.71)	2.32 (2.24–2.41)	3.53 (3.36–3.71)
Adjusted	1.51 (1.48–1.55)	1.98 (1.91–2.06)	2.79 (2.65–2.94)
**1-year ICU admission HR (95% CI)**			
Unadjusted	2.34 (2.18–2.52)	4.32 (3.95–4.72)	7.04 (6.38–7.76)
Adjusted	2.09 (1.94–2.25)	3.59 (3.28–3.92)	5.35 (4.84–5.91)
**5-year ICU admission HR (95% CI)**			
Unadjusted	1.86 (1.79–1.93)	2.92 (2.78–3.07)	4.84 (4.56–5.14)
Adjusted	1.64 (1.58–1.70)	2.42 (2.30–2.54)	3.65 (3.43–3.87)
**8-year ICU admission HR (95% CI)**			
Unadjusted	1.74 (1.69–1.79)	2.69 (2.58–2.81)	4.28 (4.05–4.52)
Adjusted	1.54 (1.49–1.59)	2.23 (2.14–2.34)	3.24 (3.06–3.42)

*HR = hazard ratio; CI = confidence interval; ICU = intensive care unit*. For all outcomes, the comparator is subjects in fit categories (n = 64,650). All data adjusted for age and gender.

**Table 3:** 1-, 5- and 8-year adjusted hazard ratios for outcomes of all-cause mortality, unplanned hospitalization and ICU admission associated with different frailty categories stratified by gender (n = 86,133).

Outcome	Mild frailty (n = 14,244)		Moderate frailty (n = 4,741)		Severe frailty (n = 2,498)	
	Male (n = 7,478)	Female (n = 6,766)	Male (n = 2,692)	Female (n = 2,049)	Male (n = 1,578)	Female (n = 920)
**All-cause mortality aHR (95% CI)**						
**1 year**	1.83 (1.65–2.04)	1.88 (1.66–2.13)	2.70 (2.37–3.07)	3.73 (3.22–4.32)	4.84 (4.26–5.49)	5.29 (4.46–6.27)
**5 year**	1.41 (1.35–1.48)	1.54 (1.46–1.62)	1.93 (1.82–2.06)	2.52 (2.34–2.71)	3.07 (2.86–3.28)	3.79 (3.46–4.15)
**8 year**	1.35 (1.30–1.41)	1.48 (1.42–1.55)	1.85 (1.75–1.95)	2.31 (2.17–2.46)	2.77 (2.61–2.94)	3.48 (3.22–3.76)
**Unplanned hospitalization aHR (95% CI)**						
**1 year**	1.87 (1.73–2.01)	1.95 (1.80–2.11)	2.73 (2.48–3.00)	3.03 (2.72–3.38)	4.24 (3.83–4.71)	4.36 (3.81–4.98)
**5 year**	1.58 (1.51–1.64)	1.66 (1.59–1.73)	2.05 (1.93–2.17)	2.28 (2.14–2.44)	3.00 (2.80–3.21)	3.16 (2.89–3.46)
**8 year**	1.48 (1.43–1.53)	1.56 (1.50–1.62)	1.91 (1.81–2.01)	2.10 (1.98–2.22)	2.76 (2.59–2.95)	2.85 (2.62–3.11)
**ICU admission aHR (95% CI)**						
**1 year**	2.02 (1.83–2.23)	2.18 (1.95–2.44)	3.28 (2.91–3.69)	4.09 (3.56–4.70)	4.85 (4.27–5.51)	6.44 (5.49–7.55)
**5 year**	1.58 (1.50–1.66)	1.73 (1.64–1.82)	2.24 (2.10–2.40)	2.70 (2.50–2.91)	3.39 (3.15–3.66)	4.21 (3.82–4.65)
**8 year**	1.48 (1.42–1.54)	1.62 (1.55–1.70)	2.05 (1.94–2.18)	2.54 (2.37–2.71)	2.99 (2.79–3.21)	3.79 (3.47–4.15)

*aHR = adjusted hazard ratio; CI = confidence interval; ICU = intensive care unit*. For all outcomes, the comparator is subjects in fit categories (n = 64,650). All data adjusted for age.

## Discussion

Previous studies used data from prospective longitudinal study[16] or health survey[17] to construct FI; however the patient numbers were still not large enough (n = 2,218 and 29,905, respectively). Besides, these studies only demonstrate the association between FI and mortality. The association between FI and other adverse outcomes remains unknown. Another study in Taiwan had same limitations mentioned above.[18] To the best of our knowledge, this is the first study to construct a frailty index using a nationwide health insurance claims database. We described the characteristics of the mFI and demonstrated its ability to predict all-cause mortality and hospitalization over an extended time period (up to 8 years). The Kaplan-Meier curves showed that the risks for mortality, unplanned hospitalization and ICU admission significantly increased with higher mFI.

A major strength of our study is that we constructed the mFI using the cumulative deficit approach based on diagnoses recorded in the large claims database. This approach guarantees the applicability of our mFI to many different settings, as many existing electronic medical databases or claims databases, such as the Medicare Coverage Database[19] and the General Practice Research Database (GPRD)[20], consist of comprehensive records of diagnoses of patients or beneficiaries. Our mFI thus could be easily developed and used for overall risk stratification, care management and healthcare resource allocation. Although we used quartile to categorize patients into 4 frailty groups, sensitivity analyses using tertile or quintile as cut point to categorize study population into 3 or 5 groups were also conducted (**S3 and S4 Tables**), which yielded similar results that the higher the eFI, the higher the risk of adverse outcomes. Still, we look forward to external validations of this mFI either using other databases or comparing the performance of this mFI with other pre-developed frailty index to further assess the generalizability and applicability of the mFI.

Another strength of our mFI is that the number of deficits included in our mFI is less than the number used in a previous study using routine primary care electronic health record data in the UK.[13] We included 32 deficits in our mFI while the UK study included 36 deficits in their frailty index. The discrepancy between the study completed in the UK and our study is that we required the prevalence of a potential deficit to be more than 2%, while the UK study included deficits with prevalence to be more than 0.5%. With fewer deficits included in our mFI, the ability of the mFI to predict risk of short- (at 1 year) and long-term (at 5 or 8 years) hospitalizations and mortality has been found to be effective. Our mFI thus could be more user-friendly and time-saving for clinical practitioners to quickly screen those who are most frail or who need the most intense intervention.

The characteristics of the mFI in our study are similar to those of other FIs developed in other nationwide samples of older adults, which include right-skewed distribution and increase with chronological age.[16, 17] However, the magnitude of the mean mFI is larger in men in this study, which is inconsistent with a recent study in Taiwan[18]. A potential explanation is that “hyperplasia of prostate” was included as a deficit in the mFI construction which is unlikely to be counted as a deficit in women. Meanwhile, the uneven distribution of some diseases between men and women may also contribute to this phenomenon.

Even though our study has the strength of demonstrating the construction of an mFI and the mFI was highly associated with all-cause mortality, unplanned hospitalization and ICU admission, it has some limitations due to the nature of claims data. First, previous studies have indicated that variables related to medical conditions, physical activity, cognitive function, health attitude and mood are all important components of frailty.[21–23] However, most of those components were not available or incompletely measured in the health insurance claims database. Nevertheless, our study did show the ability of the mFI to predict risk of hospitalization and mortality even without these components. Second, frailer people may have fewer encounters with the healthcare system, which may cause misclassification bias. Third, physicians may not reliably submit the ICD-9 diagnosis codes related to frailty, such as abnormality of gait, difficulty walking and fatigue. Therefore, the prevalence of *a prior* ICD-9-CM diagnosis codes presenting clinical manifestation of frail people[24] was low in NHIRD, which made it incompatible with inclusion as a deficit. In addition, as the deficit selection was based on the ICD-9 codes, we understand that some of the “deficits” may be combined in clinical setting. However, as we required the prevalence of any identified deficit to be more than 2%, these deficits should have representativeness in our study cohort. Lastly, we did not include diabetes mellitus, a very common disease in our list of deficit items. The reason is because that diabetes mellitus did not meet the criteria we select. While the prevalence of diabetes mellitus is 18.15% among our study cohort, the prevalence is not increasing with age, which violates one of our criterion to select deficit (after plotting the prevalence of diagnoses among 5 age groups (65–69, 70–74, 75–79, 80–84 and ≥85 years old), the linear regression coefficients should be positive and R^2^ value should be more than 0.30). However, even without including diabetes mellitus in our mFI, the ability of the mFI to predict risk of hospitalization and mortality is still good.

Despite the above-mentioned limitations, this study did provide important information on which to base further research. The ability of the mFI to predict all-cause mortality, unplanned hospitalization and ICU admission has implications for research and policy implementation. Further studies may investigate whether the mFI is a predictor of other adverse outcomes, such as falls[25] and fractures.[26] Additionally, this study measured the mFI only during a one-year baseline period. The time-varying frailty based on multiple measurements may provide in-depth information to identify high-risk subjects for the public health consideration.[27]

## Conclusions

The mFI was highly associated with all-cause mortality, unplanned hospitalization and ICU admission. It may serve as an efficient tool for stratifying older adults into care management programs and other real-world studies.

## Supporting information

S1 Table Prevalence of individual deficits in the total population and in different age groups. (DOCX)

S2 Table *C*-statistics and pseudo-R^2^ for the outcomes of all-cause mortality, unplanned hospitalization and ICU admission. (DOCX)

S3 Table Sensitivity analysis using tertile of multimorbidity frailty index as cut points to categorize study population into 3 frailty groups. (DOCX)

S4 Table Sensitivity analysis using quintile of multimorbidity frailty index as cut points to categorize study population into 5 frailty groups. (DOCX)

S1 Fig Distribution of the multimorbidity frailty index (mFI). (DOCX)
