# Supplementary material for: Predicting mortality and hospitalization of older adults by the multimorbidity frailty index
Source: PLoS One. 2017 Nov 16;12(11):e0187825. doi: 10.1371/journal.pone.0187825 (PMC5690585; doi:10.1371/journal.pone.0187825)
Supplement: S1 Table — (DOCX) [file pone.0187825.s001.docx]

# S1 Table. Prevalence of individual deficits in the total population and in different age groups

| ICD-9-CM code | Deficit | Overall | Age group (y/o) | | | | |
| --- | --- | --- | --- | --- | --- | --- | --- |
|  |  |  | 65-69 | 70-74 | 75-79 | 80-84 | ≥85 |
|  |  | n=86,133 | n=28,480 | n=23,700 | n=18,765 | n=9,934 | n=5,254 |
| 285 | Other and unspecified anemias | 2.03% | 1.18% | 1.74% | 2.46% | 3.16% | 4.26% |
| 290 | Senile and presenile organic psychotic conditions | 2.71% | 0.81% | 1.96% | 3.29% | 5.75% | 8.51% |
| 332 | Parkinson's disease | 2.08% | 0.98% | 1.96% | 2.80% | 3.65% | 3.06% |
| 374 | Other disorders of eyelids | 2.62% | 1.78% | 2.50% | 3.34% | 3.48% | 3.56% |
| 386 | Vertiginous syndromes and other disorders of vestibular system | 3.96% | 3.17% | 3.93% | 4.67% | 4.80% | 4.17% |
| 402 | Hypertensive heart disease | 14.20% | 11.10% | 14.31% | 16.57% | 17.39% | 15.93% |
| 414 | Other forms of chronic ischemic heart disease | 11.55% | 8.36% | 11.68% | 14.17% | 14.89% | 12.66% |
| 427 | Cardiac dysrhythmias | 5.87% | 3.94% | 5.57% | 7.09% | 8.45% | 8.49% |
| 428 | Heart failure | 3.97% | 2.17% | 3.42% | 4.66% | 6.75% | 8.53% |
| 434 | Occlusion of cerebral arteries | 4.71% | 3.13% | 4.51% | 5.90% | 6.66% | 6.13% |
| 437 | Other and ill-defined cerebrovascular disease | 2.31% | 1.55% | 2.07% | 2.99% | 3.27% | 3.27% |
| 438 | Late effects of cerebrovascular disease | 3.72% | 2.27% | 3.28% | 4.57% | 6.03% | 6.15% |
| 486 | Pneumonia, organism unspecified | 2.66% | 1.26% | 2.07% | 3.42% | 4.77% | 6.28% |
| 491 | Chronic bronchitis | 5.39% | 3.29% | 4.82% | 6.74% | 8.57% | 8.55% |
| 493 | Asthma | 3.90% | 3.30% | 3.88% | 4.27% | 4.62% | 4.51% |
| 496 | Chronic airways obstruction, not elsewhere classified | 4.22% | 1.97% | 3.73% | 5.63% | 7.21% | 7.94% |
| 530 | Diseases of esophagus | 2.43% | 1.89% | 2.38% | 2.74% | 3.26% | 2.91% |
| 531 | Gastric ulcer | 3.68% | 2.77% | 3.82% | 4.20% | 4.40% | 4.76% |
| 532 | Duodenal ulcer | 2.10% | 1.69% | 2.11% | 2.45% | 2.42% | 2.46% |
| 536 | Disorders of function of stomach | 7.90% | 6.76% | 7.68% | 8.89% | 9.52% | 8.41% |
| 558 | Other noninfectious gastroenteritis and colitis | 2.48% | 2.28% | 2.55% | 2.52% | 2.73% | 2.74% |
| 564 | Functional digestive disorders, not elsewhere classified | 11.72% | 7.58% | 10.65% | 14.71% | 17.20% | 17.97% |
| 585 | Chronic renal failure | 2.01% | 1.62% | 2.05% | 2.22% | 2.53% | 2.17% |
| 599 | Other disorders of urethra and urinary tract | 6.11% | 4.10% | 5.52% | 7.11% | 9.16% | 10.32% |
| 600 | Hyperplasia of prostate | 8.58% | 5.42% | 8.30% | 11.76% | 11.92% | 9.25% |
| 682 | Other cellulitis and abscess | 2.16% | 1.59% | 2.00% | 2.61% | 2.89% | 2.97% |
| 692 | Contact dermatitis and other eczema | 7.41% | 5.76% | 6.95% | 8.98% | 9.39% | 9.06% |
| 698 | Pruritus and related conditions | 3.26% | 2.66% | 3.17% | 3.84% | 3.86% | 3.69% |
| 733 | Other disorders of bone and cartilage | 5.15% | 3.94% | 5.08% | 5.74% | 6.53% | 7.23% |
| 780 | General symptoms | 19.80% | 16.92% | 19.54% | 22.24% | 23.05% | 21.60% |
| 785 | Symptoms involving cardiovascular system | 2.15% | 1.91% | 2.21% | 2.19% | 2.49% | 2.34% |
| 788 | Symptoms involving urinary system | 3.18% | 2.24% | 3.28% | 3.98% | 3.93% | 3.58% |
