# Supplementary material for: Predicting mortality and hospitalization of older adults by the multimorbidity frailty index
Source: PLoS One. 2017 Nov 16;12(11):e0187825. doi: 10.1371/journal.pone.0187825 (PMC5690585; doi:10.1371/journal.pone.0187825)
Supplement: S2 Table — (DOCX) [file pone.0187825.s002.docx]

# S2 Table. *C*-statistics and pseudo-R^2^ for the outcomes of all-cause mortality, unplanned hospitalization and ICU admission

| Outcomes | 1 year | | 5 year | | 8 year | |
| --- | --- | --- | --- | --- | --- | --- |
|  | *C* (95% CI) | R^2^ | *C* (95% CI) | R^2^ | *C* (95% CI) | R^2^ |
| **All-cause mortality** | 0.6697 (0.6603-0.6791) | 0.0200 | 0.6253 (0.6207-0.6298) | 0.0467 | 0.6203 (0.6164-0.6242) | 0.0546 |
| **Unplanned hospitalization** | 0.6516 (0.6451-0.6581) | 0.0253 | 0.6154 (0.6114-0.6193) | 0.0353 | 0.5973 (0.5936-0.6009) | 0.0262 |
| **ICU admission** | 0.6791 (0.6706-0.6876) | 0.0220 | 0.6279 (0.6233-0.6325) | 0.0375 | 0.6098 (0.6058-0.6139) | 0.0339 |

*ICU= intensive care unit*
