# Supplementary material for: Predicting mortality and hospitalization of older adults by the multimorbidity frailty index
Source: PLoS One. 2017 Nov 16;12(11):e0187825. doi: 10.1371/journal.pone.0187825 (PMC5690585; doi:10.1371/journal.pone.0187825)
Supplement: S3 Table — (DOCX) [file pone.0187825.s003.docx]

# S3 Table. Sensitivity analysis using tertile of multimorbidity frailty index as cut points to categorize study population into 3 frailty groups

| Outcome | 0.083<eFI<=0.167  (n=17,241) | eFI>0.167 (n=4,242) |
| --- | --- | --- |
| **1-year all-cause mortality HR (95% CI)** | | |
| Unadjusted | 2.46 (2.28-2.64) | 6.40 (5.88-6.98) |
| Adjusted | 2.02 (1.88-2.18) | 4.42 (4.05-4.82) |
| **5-year all-cause mortality HR (95% CI)** | | |
| Unadjusted | 1.90 (1.84-1.96) | 4.23 (4.05-4.42) |
| Adjusted | 1.55 (1.50-1.60) | 2.92 (2.79-3.06) |
| **8-year all-cause mortality HR (95% CI)** | | |
| Unadjusted | 1.81 (1.76-1.86) | 3.82 (3.68-3.97) |
| Adjusted | 1.48 (1.45-1.52) | 2.67 (2.57-2.77) |
| **1-year unplanned hospitalization HR (95% CI)** | | |
| Unadjusted | 2.24 (2.13-1.91) | 4.62 (4.32-4.94) |
| Adjusted | 2.03 (1.93-2.14) | 3.82 (3.57-4.09) |
| **5-year unplanned hospitalization HR (95% CI)** | | |
| Unadjusted | 1.87 (1.82-1.92) | 3.36 (3.22-3.51) |
| Adjusted | 1.68 (1.63-1.72) | 2.75 (2.63-2.87) |
| **8-year unplanned hospitalization HR (95% CI)** | | |
| Unadjusted | 1.75 (1.71-1.79) | 3.08 (2.96-3.21) |
| Adjusted | 1.57 (1.53-1.61) | 2.51 (2.41-2.62) |
| **1-year ICU admission HR (95% CI)** | | |
| Unadjusted | 2.60 (2.43-2.78) | 6.22 (5.72-6.75) |
| Adjusted | 2.29 (2.14-2.45) | 4.86 (4.46-5.29) |
| **5-year ICU admission HR (95% CI)** | | |
| Unadjusted | 1.98 (1.92-2.05) | 4.22 (4.02-4.43) |
| Adjusted | 1.73 (1.67-1.79) | 3.29 (3.13-3.45) |
| **8-year ICU admission HR (95% CI)** | | |
| Unadjusted | 1.85 (1.79-1.90) | 3.76 (3.60-3.93) |
| Adjusted | 1.62 (1.57-1.67) | 2.95 (2.82-3.09) |

*HR=hazard ratio; CI=confidence interval; ICU= intensive care unit*

For all outcomes, the comparator is subjects with 0<eFI<=0.083 (n=64,650).

All data adjusted for age and gender.
