# Supplementary material for: Predicting mortality and hospitalization of older adults by the multimorbidity frailty index
Source: PLoS One. 2017 Nov 16;12(11):e0187825. doi: 10.1371/journal.pone.0187825 (PMC5690585; doi:10.1371/journal.pone.0187825)
Supplement: S4 Table — (DOCX) [file pone.0187825.s004.docx]

# S4 Table. Sensitivity analysis using quintile of multimorbidity frailty index as cut points to categorize study population into 5 frailty groups

| Outcome | 0.05<eFI<=0.10  (n=23,521) | 0.10<eFI<=0.15 (n=5,253) | 0.15<eFI<=0.20  (n=4,741) | eFI>0.20  (n=2,498) |
| --- | --- | --- | --- | --- |
| **1-year all-cause mortality HR (95% CI)** | | | |  |
| Unadjusted | 1.81 (1.67-1.96) | 2.95 (2.63-3.30) | 4.69 (4.16-5.09) | 8.45 (7.62-9.38) |
| Adjusted | 1.58 (1.46-1.72) | 2.31 (2.06-2.59) | 3.38 (3.06-3.75) | 5.46 (4.91-6.07) |
| **5-year all-cause mortality HR (95% CI)** | | | |  |
| Unadjusted | 1.50 (1.45-1.56) | 2.28 (2.17-2.40) | 3.09 (2.94-3.24) | 5.42 (5.13-5.73) |
| Adjusted | 1.30 (1.26-1.35) | 1.77 (1.68-1.86) | 2.26 (2.16-2.38) | 3.47 (3.28-3.67) |
| **8-year all-cause mortality HR (95% CI)** | | | |  |
| Unadjusted | 1.48 (1.44-1.52) | 2.14 (2.05-2.23) | 2.86 (2.75-2.98) | 4.85 (4.63-5.09) |
| Adjusted | 1.28 (1.24-1.31) | 1.67 (1.60-1.74) | 2.12 (2.03-2.21) | 3.14 (2.99-3.30) |
| **1-year unplanned hospitalization HR (95% CI)** | | | |  |
| Unadjusted | 1.81 (1.71-1.91) | 2.81 (2.60-3.04) | 3.75 (3.48-4.04) | 6.01 (5.54-6.53) |
| Adjusted | 1.69 (1.60-1.79) | 2.49 (2.30-2.70) | 3.22 (2.98-3.47) | 4.84 (4.45-5.26) |
| **5-year unplanned hospitalization HR (95% CI)** | | | |  |
| Unadjusted | 1.61 (1.57-1.66) | 2.26 (2.16-2.36) | 2.77 (2.65-2.89) | 4.26 (4.03-4.50) |
| Adjusted | 1.50 (1.46-1.54) | 1.98 (1.89-2.06) | 2.35 (2.24-2.45) | 3.35 (3.17-3.54) |
| **8-year unplanned hospitalization HR (95% CI)** | | | |  |
| Unadjusted | 1.54 (1.50-1.57) | 2.07 (1.99-2.15) | 2.54 (2.44-2.64) | 3.86 (3.67-4.06) |
| Adjusted | 1.43 (1.40-1.46) | 1.81 (1.74-1.88) | 2.15 (2.06-2.23) | 3.03 (2.87-3.19) |
| **1-year ICU admission HR (95% CI)** | | | |  |
| Unadjusted | 1.98 (1.84-2.14) | 3.39 (3.06-3.76) | 5.04 (4.59-5.54) | 8.22 (7.42-9.10) |
| Adjusted | 1.82 (1.69-1.96) | 2.90 (2.62-3.22) | 4.14 (3.76-4.55) | 6.18 (5.56-6.86) |
| **5-year ICU admission HR (95% CI)** | | | |  |
| Unadjusted | 1.64 (1.59-1.70) | 2.42 (2.29-2.55) | 3.24 (3.08-3.42) | 5.38 (5.06-5.71) |
| Adjusted | 1.49 (1.44-1.55) | 2.05 (1.94-2.16) | 2.64 (2.51-2.78) | 4.00 (3.76-4.25) |
| **8-year ICU admission HR (95% CI)** | | | |  |
| Unadjusted | 1.58 (1.53-1.62) | 2.19 (2.09-2.29) | 2.96 (2.83-3.10) | 4.70 (4.45-4.97) |
| Adjusted | 1.44 (1.40-1.48) | 1.86 (1.78-1.95) | 2.42 (2.32-2.54) | 3.52 (3.32-3.72) |

*HR=hazard ratio; CI=confidence interval; ICU= intensive care unit*

For all outcomes, the comparator is subjects with 0<eFI<=0.05 (n=50,120).

All data adjusted for age and gender.
