# Supplementary material for: Predicting mortality and hospitalization of older adults by the multimorbidity frailty index
Source: PLoS One. 2017 Nov 16;12(11):e0187825. doi: 10.1371/journal.pone.0187825 (PMC5690585; doi:10.1371/journal.pone.0187825)
Supplement: S1 Fig — (DOCX) [file pone.0187825.s005.docx]

# S1 Fig. Distribution of the multimorbidity frailty index (mFI)
